# Regional anesthesia for hysterectomies across a large healthcare system: location matters

**DOI:** 10.1186/s12871-026-04083-5

**Published:** 2026-07-15

**Authors:** Naomi Goldstein, Liam Sasser, Nicholas A. Giordano, Alexander Velosky, Michael S. Patzkowski, Krista B. Highland

**Affiliations:** 1 Department of Obstetrics and Gynecology, The Ohio State University Wexner Medical Center, Columbus, OH USA; 2 Sanford I. Weill Medical College, Cornell University, New York City, NY USA; 3 Nell Hodgson Woodruff School of Nursing, Emory University, Atlanta, GA USA; 4 Defense and Veterans Consortium for Integrative Pain Management, Department of Anesthesiology, Uniformed Services University, Bethesda, MD USA; 5 Henry M. Jackson Foundation for the Advancement of Military Medicine, Inc., Bethesda, MD USA; 6 Department of Anesthesiology, Uniformed Services University, Bethesda, MD USA; 7 Department of Anesthesia, Brooke Army Medical Center, Fort Sam Houston, TX USA

**Keywords:** Gynecological surgery, Pain management, Adjunctive medications, Multimodal anesthesia

## Abstract

**Purpose:**

While guidelines emphasize the benefits of multimodal anesthesia for hysterectomy procedures, including regional anesthesia (RA), utilization rates remain understudied. This study described multilevel factors associated with RA receipt across the US Military Health System.

**Methods:**

This retrospective cohort study included patients undergoing a hysterectomy between January 2022 and April 2025 (*N* = 5,150). Patient- (e.g., demographic and clinical information), care- (e.g., route, surgical duration, adjuncts), and system- (e.g., facility) level factors were covariates in a generalized additive mixed-effects model (GAMM) evaluating RA receipt.

**Results:**

Overall, 13% received RA. Across 33 facilities, RA use ranged from 0 to 66% (median 9%, IQR 3–15%). In the GAMM, surgical facility accounted for 23% of the outcome variance. Odds (95% CI) of RA receipt were higher for patients with a Charlson Comorbidity Index of 1 relative to 0 (OR = 1.33, 1.03–1.71, *p* = 0.03), with leiomyoma diagnoses (OR = 1.26, 1.01–1.58, *p* = 0.04), and who received an open hysterectomy (OR = 6.02, 4.55–7.96, *p* < 0.001) and more adjuncts (OR = 1.15, 1.05–1.27, *p* = 0.005). The smooth effect of age was significant (*p* = 0.03), such increasing age was associated with lower odds of RA receipt.

**Conclusion:**

RA receipt was not common; variability was largely attributed to facility. Patients receiving RA tended to receive more adjuncts (e.g., dexmedetomidine, ketamine, NSAID, acetaminophen, gabapentinoids), which suggests anesthesia clinicians who use RA are also those who more frequently use multimodal analgesia. Further research is needed to evaluate acute and subacute hysterectomy outcomes that accounts for the full range of anesthesia and analgesia selection, as well as clinician practice patterns and motivations for RA use.

## Introduction

 Multimodal analgesia is a key component of the American College of Obstetricians and Gynecologists Enhanced Recovery After Surgery (ERAS) recommendations for hysterectomies [1], to include regional anesthesia (RA), local anesthetics (e.g., port site, subcutaneous, subfascial, or intraperitoneal infiltration) and adjunct medications (e.g., acetaminophen, gabapentinoids, and nonsteroidal anti-inflammatory drugs (NSAIDs), including COX-2 inhibitors). Evidence indicates additional perioperative adjunct medications may support improved outcomes after hysterectomy, such as dexmedetomidine [2] and ketamine [3–5]. Given guideline-aligned practices, such as RA receipt, vary across patient-, care-, and system- (e.g., geographic region) level factors [6–8]. evaluating practice patterns across large healthcare systems could enable wide-scale efforts to optimize anesthesia and analgesia for hysterectomies.

The US Military Health System (MHS) includes a network of 45 hospitals and 572 ambulatory care and occupational health facilities located across the globe. Each year, millions of active duty service members, retired service members, and their family members receive healthcare at these military treatment facilities. As of 2020, MHS operations became centrally managed by the Defense Health Agency. Thus, there is an opportunity to reduce unwarranted variation and ensure strategic personnel allocation, while also optimizing engagement in critical combat casualty care skills, such as RA, per the Joint Trauma System clinical practice guidelines [9]. Given the ubiquity of hysterectomies within the US [10], evaluating RA and adjunct receipt for hysterectomies represents a meaningful use case for enterprise-wide monitoring and process improvement. The goal of this study was to evaluate RA receipt within the MHS, to include patient-, care-, and system-level factors associated with use, with particular emphasis on between-facility variation. It was hypothesized that patient-level factors (e.g., reason for surgery), care-level factors (e.g., adjuncts), and system-level factors (e.g., facility) would be associated with RA receipt.

## Methods

### Data Sources and Record Selection

This retrospective study received an exempt determination by the Uniformed Services University Institutional Review Board. Data was sourced from the MHS Information Platform and included both claims data and structured field entries from the electronic health record, Genesis. Patient surgical encounter records were included for any patient who received a hysterectomy between January 1, 2022 and April 16, 2025 at a military treatment facility without epidural or spinal anesthesia (*N* = 5,981). Patient records were excluded if a chest surgery or child birth occurred in the same encounter (*n* = 12), there was a return to the operating room (*n* = 20), discrepant hysterectomy approaches were recorded (*n* = 113), clear documentation of surgery and anesthesia start and stop/extubation times was lacking, including administration of muscle relaxants and reversal medications (e.g., sugammadex) to indicate general anesthesia was used (*n* = 63), patients were not enrolled in Direct Care Only or TRICARE Prime plans (*n* = 399), the surgical duration was in the 1st or 99th percentile (*n* = 118), and patients received a hysterectomy at a facility with 20 or fewer documented hysterectomies (*n* = 106), as analyses required sufficient sample size within facility. Therefore, 5,150 patient records were included in the analytic sample.

### Primary outcome

Receipt of perioperative RA was the primary outcome. Information regarding perioperative RA was obtained by evaluating structured health record data fields indicating “regional block start” and/or “perineural” medication administration times prior to extubation, as anesthesia procedures are not itemized in claims data and therefore, Current Procedural Terminology (CPT) codes were not available. Due to inconsistent variation in recorded block type and lack of analyzable and accessible free text data, details regarding the type of RA (e.g., transverse abdominal plane block) were not known. Any RA occurring after extubation were not included, as post-procedural administration was indicative of care delivered by the acute pain service for emergent pain management requirements.

### Potential covariates

Potential covariates included patient-, care-, and system-level information. Patient-level information included age, race and ethnicity, beneficiary category (active duty service member, family member, another beneficiary type), age-adjusted Charlson Comorbidity Index (CCI) [11], and past 6-month documented International Classification of Diseases - Version 10 (ICD-10) codes corresponding to psychiatric conditions, obesity diagnosis, urinary conditions, non-gynecological pain conditions per prior literature [12], pelvic pain, abnormal or excessive menstrual bleeding, non-inflammatory gynecological conditions, inflammatory gynecological conditions, menstrual pain, leiomyomas, gynecological neoplasm, endometriosis, peritoneum disorder, and oncological prophylaxis requirements. CCI scores were categorized as 0, 1, and 2+. Care-level information included hysterectomy route (minimal versus open) identified via Current Procedure Terminology (CPT) and ICD-10 Procedure Coding System (PCS) codes. Additional care-level information included surgical duration and number of adjuncts (acetaminophen, gabapentinoids, NSAIDs, dexmedetomidine, ketamine). System-level information included surgical facility and year.

### Analytic plan

Non-parametric bivariate analyses compared patients who did not and did receive perioperative RA using the *CompareGroups* R package [13]. Calculated with the *cobalt* R package [14], included covariates were those with a standardized mean difference between groups > 0.10. A generalized additive mixed-effects model (GAMM) with a binomial outcome distribution evaluated the relationship between covariates and perioperative RA receipt. In a GAMM, continuous covariates with adequate range can be modeled as nonlinear smooth terms. The GAMM was estimated with the *mgcv* R package [15] first without and then with a random effect for facility; the variance explained (R-squared) was then compared between models. GAMM results were tabulated using the *sjPlot* [16] and *ggeffects* [17] R packages. Results are displayed using the *ggplot2* R package [18].

## Results

Of the 5,150 patient surgical encounters (91% minimally invasive procedures) included in the study, 679 (13.2%; 11% minimally invasive, 37% open) received RA before extubation. Of those receiving RA, “perineural” medication administration (86%) was more often documented than “regional block start” times (70%). Standardized mean differences greater than the 0.10 threshold included patient age, race and ethnicity, beneficiary category, age-adjusted CCI category, sleep apnea diagnosis, several reasons for hysterectomy (abnormal or excessive menstrual bleeding, inflammatory gynecological condition, leiomyomas, gynecological neoplasm, peritoneum condition), hysterectomy type, number of adjuncts, and surgical duration (Table 1). These variables were included as covariates in the primary analysis.

**Table 1:** Overall sample description and bivariate differences between patients who did not (No) and did (Yes) receive regional anesthesia

	Full Sample (*N* = 5150)	No (*n* = 4471)	Yes (*n* = 679)	*p*-value
Age	42 [36;47]	41 [36;47]	43 [37;49]	< 0.01
Race & Ethnicity				< 0.01
American Indian & Alaska Native	40 (1%)	* (< 1%)	* (< 1%)	
Asian	196 (4%)	162 (4%)	34 (5%)	
Black	990 (19%)	803 (18%)	187 (28%)	
Hispanic	559 (11%)	501 (11%)	58 (9%)	
Pacific Islander	151 (3%)	* (3%)	* (2%)	
Unknown	689 (13%)	620 (14%)	69 (10%)	
White	2525 (49%)	2213 (49%)	312 (46%)	
Beneficiary Category				< 0.01
Active Duty	1131 (22%)	960 (21%)	171 (25%)	
Family Member	3590 (70%)	3181 (71%)	409 (60%)	
Other	429 (8%)	330 (7%)	99 (15%)	
ASA Level				0.15
I or II	4383 (89%)	3816 (89%)	567 (87%)	
III or IV	551 (11%)	467 (11%)	84 (13%)	
Age-Adjusted Charlson Comorbidity Index				< 0.01
0	3360 (65%)	2972 (66%)	388 (57%)	
1	1073 (21%)	908 (20%)	165 (24%)	
2+	717 (14%)	591 (13%)	126 (19%)	
Year	2023 [2023;2024]	2023 [2023;2024]	2023 [2023;2024]	0.09
Hysterectomy Type				< 0.01
Minimal	4664 (91%)	4166 (93%)	498 (73%)	
Open	486 (9%)	305 (7%)	181 (27%)	
*Pre-Hysterectomy Diagnoses*				
Nicotine Dependence & Tobacco Use	469 (9%)	407 (9%)	62 (9%)	1.00
Pain-Related Diagnosis	3859 (75%)	3344 (75%)	515 (76%)	0.59
Psychiatric Diagnosis	1954 (38%)	1676 (37%)	278 (41%)	0.09
Obesity Diagnosis	1060 (21%)	905 (20%)	155 (23%)	0.13
Gastroesophageal Reflux Disease	574 (11%)	495 (11%)	79 (12%)	0.71
Primary Hypertension	994 (19%)	851 (19%)	143 (21%)	0.23
Sleep Apnea	516 (10%)	422 (9%)	94 (14%)	< 0.01
High Cholesterol	628 (12%)	537 (12%)	91 (13%)	0.33
Pelvic Pain	1249 (24%)	1076 (24%)	173 (25%)	0.45
Menstrual Pain	1922 (37%)	1675 (37%)	247 (36%)	0.62
Leiomyoma	2182 (42%)	1819 (41%)	363 (53%)	< 0.01
Abnormal/Excessive Menstrual Bleeding	3896 (76%)	3421 (77%)	475 (70%)	< 0.01
Inflammatory Gynecological Condition	901 (17%)	748 (17%)	153 (23%)	< 0.01
Endometriosis	1490 (29%)	1287 (29%)	203 (30%)	0.58
Urinary Diagnosis	901 (17%)	790 (18%)	111 (16%)	0.43
Non-Inflammatory Gynecological Condition	3401 (66%)	2944 (66%)	457 (67%)	0.48
Peritoneum Disorder	358 (7%)	290 (6%)	68 (10%)	< 0.01
Gynecological Neoplasm	291 (6%)	238 (5%)	53 (8%)	0.01
Prophylactic Procedure	593 (12%)	528 (12%)	65 (10%)	0.10
*Perioperative Information*				
Adjuncts	3 [2;3]	3 [2;3]	3 [2;4]	< 0.01
Opioid	5061 (98%)	4397 (98%)	664 (98%)	0.38
Acetaminophen	4722 (92%)	4085 (91%)	637 (94%)	0.04
NSAID	2928 (57%)	2507 (56%)	421 (62%)	< 0.01
Dexmedetomidine	2012 (39%)	1736 (39%)	276 (41%)	0.39
Dexamethasone	5003 (97%)	4335 (97%)	668 (98%)	0.05
Ketamine	1589 (31%)	1390 (31%)	199 (29%)	0.37
Gabapentinoid	2778 (54%)	2334 (52%)	444 (65%)	< 0.01
Surgical Duration (Minutes)	145 [112;190]	144 [111;187]	157 [120;210]	< 0.01

*Values were obfuscated due to a cell size < 11

**Fig. 1:**
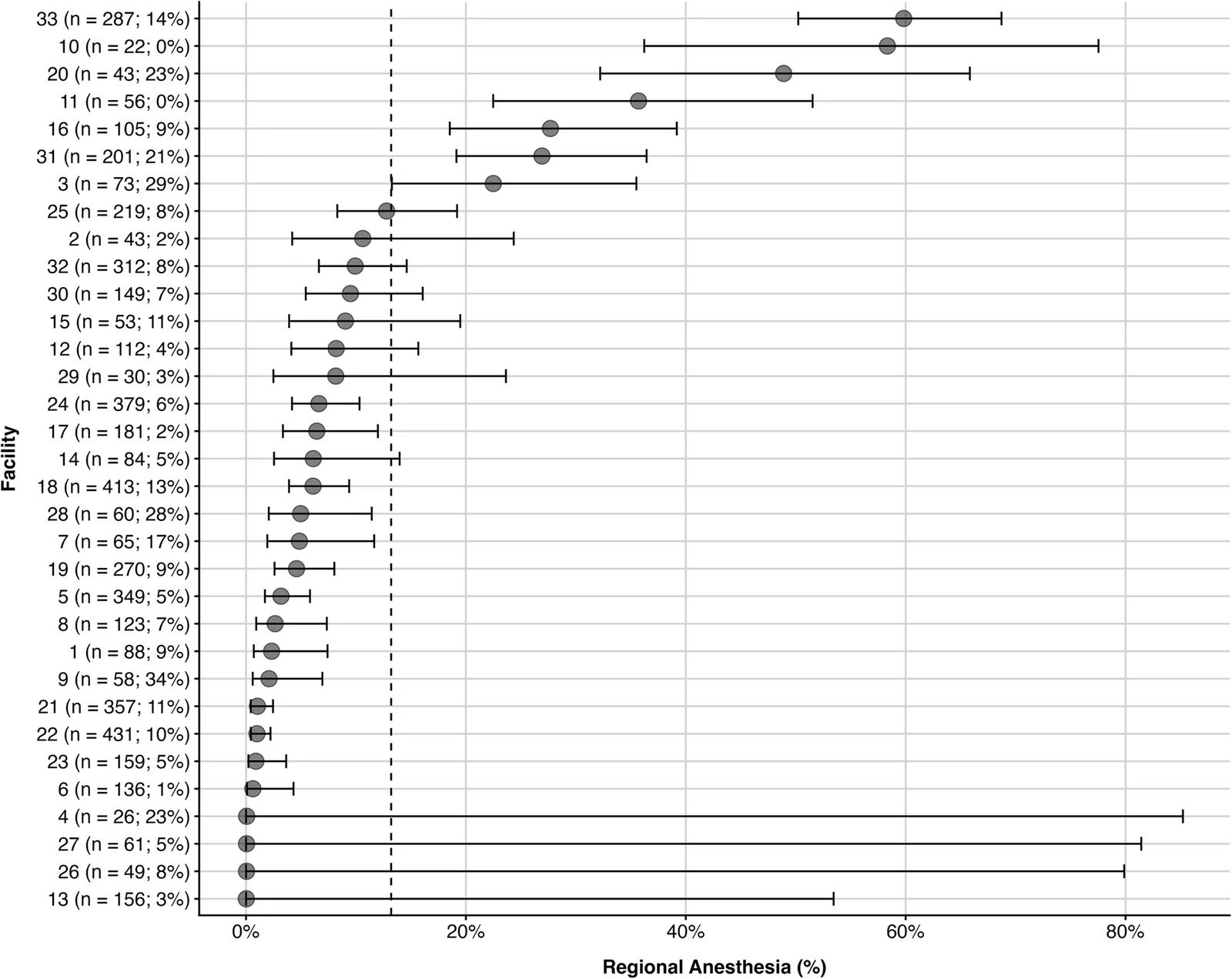
Estimated mean probabilities (95% CI) of regional anesthesia receipt by facility. Each facility is described as rank for volume (1 = highest volume, 33 = lowest volume), volume, and proportion of hysterectomies with an open approach. Estimations are derived from the random effect for facility in the generalized additive mixed-effects model. Dashed vertical line indicates overall proportion of patients receiving regional anesthesia

In the GAMM (Table 2), the outcome variance accounted for increased from 7.6% to 30.2% after including the random effect for facility. Odds (95% CI) of RA receipt were higher for patients with a CCI of 1 relative to 0 (OR = 1.33, 1.03-1.71, *p* = 0.03), with leiomyoma diagnoses (OR = 1.26, 1.01–1.58, *p* = 0.04), and who received an open hysterectomy (OR = 6.02, 4.55–7.96, *p* < 0.001) and more adjuncts (OR = 1.15, 1.05–1.27, *p* = 0.005). The smooth effect of age was significant (*p* = 0.03), such increasing age was associated with lower odds of RA receipt. For example, the predicted probability (95% CI) of RA receipt was 14% (13–15%) for a 30-year-old patient and 11% (9–13%) for a 65-year-old patient. The random effect for facility was also significant (*p* < 0.001). Variation by site is depicted in Fig. 1.

**Table 2:** Results of the generalized additive mixed-effects model predicting regional anesthesia receipt

Predictors	Odds Ratios	CI	*p*
Race & Ethnicity (Ref. White)			
American Indian & Alaska Native	0.58	0.14–2.44	0.46
Asian	1.55	0.94–2.53	0.08
Black	0.82	0.62–1.08	0.15
Hispanic	0.96	0.68–1.36	0.82
Pacific Islander	0.79	0.41–1.51	0.47
Unknown	0.74	0.53–1.04	0.09
Beneficiary Category (Ref. Active Duty Service Member)			
Family Member	0.86	0.66–1.12	0.27
Other	1.19	0.83–1.70	0.34
Age-Adjusted Charlson Comorbidity Index (Ref. 0)			
1	1.33	1.03–1.71	0.03
2+	1.17	0.83–1.64	0.36
Open Approach (Ref. Minimally Invasive)	6.02	4.55–7.96	**< 0.001**
Sleep Apnea	1.11	0.81–1.51	0.52
Leiomyoma	1.26	1.01–1.58	0.04
Abnormal/Excessive Menstrual Bleeding	0.98	0.77–1.25	0.88
Inflammatory Gynecological Condition	1.13	0.88–1.45	0.33
Gynecological Neoplasm	1.02	0.65–1.59	0.94
Peritoneum Disorder	1.18	0.83–1.68	0.36
Adjuncts	1.15	1.05–1.27	0.01
Age (Non-Linear)			0.03
Surgical Duration (Non-Linear)			0.06
Facility (Random Effect)			**< 0.001**

## Discussion

In this study of patients receiving hysterectomy with general anesthesia, approximately 13% of the sample received RA. More than one-third of patients undergoing open procedures received RA (37%), compared to 11% of those undergoing minimally invasive procedures. However, rates of RA appear to be much higher than those reported for total abdominal hysterectomy (~ 1%) in a national hospital database [6]. Moreover, a large proportion of variance in RA receipt was associated with where the patient received a hysterectomy. Such findings are similar to prior studies, which found hospital-level variation accounted for more variance in RA receipt than patient-level factors [8, 19]. Unlike prior studies, this study evaluated a single, large healthcare system that has centrally-managed monitoring, policy development, and implementation efforts. Taken together, such variation suggests an opportunity for targeted quality improvement efforts, managed by the Defense Health Agency, that could both reduce variability and enhance care.

Research has consistently reported that patient-, clinician-, and facility-level factors may influence RA use and other anesthetic approaches. Similar to the few prior studies investigating patient factors associated with RA receipt during vaginal or hysterectomy surgery, this study found that patients presenting with some comorbidities and exposed to differing surgical techniques had a greater likelihood of receiving RA [20, 21]. Specifically, in the current study, patients presenting with some comorbidities (but not significant comorbidity burden), individuals with leiomyoma, those who received open hysterectomy, and those of younger age had a greater likelihood of RA receipt. This is of concern considering some of these factors have been associated with increased incidence of moderate-severe postsurgical pain, increased length of stay, and greater postoperative complications [22, 23]. As such, future pragmatic research is warranted to discern postoperative complications risks based on the anesthesia technique utilized during hysterectomy, particularly given the infrequent use of RA seen in this study and previous investigations and need for real-world evidence.

Overall, 98% of patients received at least one adjunct and 87% received at least two. Adjuncts specified by ACOG guidance [1] were provided in most procedures (92% acetaminophen, 57% NSAID, 54% gabapentinoids), while ketamine (31%) and dexmedetomidine (39%) were also used with regular frequency. Patients who received more adjuncts were more likely to receive RA. This pattern suggests that anesthesia clinicians who use RA may vary from those who do not. Given this study did not have access to clinician information, it is unclear the extent to which clinicians who use more adjuncts are also those who are more inclined to use RA. Future research could evaluate practice patterns and motivation for practices (e.g., balancing pain management benefits and adverse events) at the anesthesia clinician-level to inform targeted practice improvement efforts. While a Defense Health Agency policy could target guideline-congruent anesthesia and analgesia, policies and CPGs are not enough to create practice change. For example, a Government Accountability Office report indicated that the MHS and Veterans Health Administration had a wealth of clinical practice guidelines, but their implementation continues to lag [24]. Previous evidence indicates that implementation-science programming, such as those to reduce postpartum hemorrhage [25], can be effectively deployed by the Defense Health Agency to improve high-value, optimized care delivery and outcomes. Overall, targeted performance improvement efforts, rooted within implementation science, could be leveraged to enhance RA access *and* evaluate patient-centered outcomes.

This study provided a baseline for future real-world evaluation of various RA approaches, as specific approaches were outside the scope of this study and location of RA administration was not reliably available. This lack of RA location data is a limitation of this study and aggregation of diverse RA interventions into a single outcome measure makes our conclusions broad and non-specific. This heterogeneity may have obscured meaningful differences in RA techniques across institutions and providers, thereby limiting the interpretability of observed practice variation. Meta-analytic findings indicate efficacy of preemptive local anesthetic infiltration for vaginal hysterectomies [26] and gynecological laparoscopic procedures [27]. However, meta-analyses indicate inconsistent evidence for transverse abdominis plane blocks for laparoscopic and robotic hysterectomies [28], but indicate erector spinae and plane and quadratus lumborum blocks may be effective for reducing pain and postoperative opioid requirements after total abdominal hysterectomy [29]. Another meta-analysis indicated the superior hypogastric plexus block may effectively reduce pain intensity and opioid requirements within the first 12 h post-hysterectomy [30]. Lastly, paracervical blocks have also shown efficacy in reducing postoperative pain and opioid requirements in the first 60 min after laparoscopic hysterectomies [31]. To enhance clarity and real-world evaluation of various anesthesia and analgesia approaches, enhanced standardization of electronic health record documentation and subsequent availability of such data for researchers may be required.

In addition to lacking information regarding specific RA approaches, this study is tempered with limitations, such as a potential lack of documentation for all RA activities. Similar to other investigations utilizing large data repositories to study anesthesia type receipt in surgical patient populations, this study’s retrospective and observational design makes the analysis vulnerable to unmeasured confounding, incomplete documentation, and documentation errors; causal conclusions are not possible. The data source utilized in this analysis does not contain details of patient or surgeon preference regarding anesthesia type, a vital factor contributing to variation in care. Moreover, additional covariates, such as underlying disease severity, case complexity, clinician training, institutional protocols, patient declination of RA, and surgical workflows, were not available and may have impacted RA use. Such information could be helpful in identifying mediators of RA use to better understand underlying factors motivating its use. The generalizability of this sample is limited. Due to the inclusion of RA in combat-relevant clinical practice guidelines, there may be elevated salience of this approach, which may be different than other healthcare systems. This study included only patients receiving a hysterectomy under general anesthesia. Some case series indicate that some minimally invasive hysterectomies may be performed with RAalone [32], and therefore, future work is needed to evaluate the extent to which this scenario occurs within the MHS and why. One registry-based study found that from 2014 to 2019, only 3% of vaginal hysterectomies were performed without general anesthesia [20]. Lastly, information regarding RA placement attempts and success were not known. Such information is critical to ensuring high-quality RA across the MHS. Despite these limitations, this study is among the first to elucidate trends in RA use among MHS beneficiaries and contributes to the nascent literature examining RA use among patients receiving hysterectomies.

## Conclusions

Overall, this study demonstrated variability in RA receipt across a large healthcare system, such that one in seven to eight patients received perioperative RA for hysterectomies, with variability across facilities. Moreover, practice patterns indicated patients receiving RA received more adjuncts, which may be due to variation in analgesic protocols (or lack thereof), tailored treatment selection, clinician training, and adjunct accessibility. Further research is needed to evaluate optimized analgesia selection and clinician practice patterns, within the context of RA receipt, that accounts for clinical characteristics that alter the risk: benefit ratio.

## Data Availability

Data from this study was obtained through a data sharing agreement with the Defense Health Agency Privacy Office and cannot be shared.
